# A New Strategy to Improve the Toughness of Epoxy Thermosets—By Introducing Poly(ether nitrile ketone)s Containing Phthalazinone Structures

**DOI:** 10.3390/ma16072878

**Published:** 2023-04-04

**Authors:** Hongjun Guo, Bing Wang, Xin Fu, Nan Li, Guiyang Li, Guodong Zheng, Zaiyu Wang, Cheng Liu, Yousi Chen, Zhihuan Weng, Shouhai Zhang, Xigao Jian

**Affiliations:** 1 State Key Laboratory of Fine Chemicals, Frontier Science Center for Smart Materials, Dalian University of Technology, Dalian 116024, China; 2 Technology Innovation Center of High Performance Resin Materials, Dalian 116024, China; 3 Aerospace Research Institute of Materials & Processing Technology, Beijing 100076, China; 4 School of Materials Science and Engineering, Dalian University of Technology, Dalian 116024, China; 5 Wuhan Second Ship Design and Research Institute, Wuhan 430064, China; 6 AVIC Jiangxi Hongdu Aviation Industry Group Company Ltd., Nanchang 330024, China

**Keywords:** PPENK, epoxy resin, toughness and strength, low-temperature properties

## Abstract

As high brittleness limits the application of all epoxy resins (EP), here, it can be modified by high-performance thermoplastic poly(ether nitrile ketone) containing phthalazinone structures (PPENK). Therefore, the influence of different PPENK contents on the mechanical, thermal, and low-temperature properties of EP was comprehensively investigated in this paper. The binary blend of PPENK/EP exhibited excellent properties due to homogeneous mixing and good interaction. The presence of PPENK significantly improved the mechanical properties of EP, showing 131.0%, 14.2%, and 10.0% increases in impact, tensile, and flexural strength, respectively. Morphological studies revealed that the crack deflection and bridging in PPENK were the main toughening mechanism in the blend systems. In addition, the PPENK/EP blends showed excellent thermal and low-temperature properties (−183 °C). The glass transition temperatures of the PPENK/EP blends were enhanced by approximately 50 °C. The 15 phr of the PPENK/EP blends had a low-temperature flexural strength of up to 230 MPa, which was 46.5% higher than EP. Furthermore, all blends exhibited better thermal stability.

## 1. Introduction

Epoxy resins are widely used in aerospace, vehicles, ships, coatings, electronic materials, and other fields due to their excellent chemical stability, heat resistance, dimensional stability, and good processing properties [1,2,3]. However, due to a large number of reactive groups in epoxy resin, it shows shortcomings such as high cross-linking density, sizeable internal stress, brittleness, poor toughness, easy cracking, and poor impact resistance after curing [4]. This limits its wide applications in structural adhesives, wind power blades, electronic and electrical packaging materials, and high-performance composites for aerospace. Therefore, modifying epoxy resin, especially improving its toughness, has always been an important research direction for researchers [5].

Researchers have extensively studied the toughening modification of epoxy resin in the past few decades, and there are mainly two types of methods. One is introducing a “flexible segment” [6,7]. By modifying the curing agent or epoxy resin, the “flexible segment” enters, thereby improving the resin molecular chain’s flexibility, fluidity, and toughness. However, it significantly reduces the strength and modulus of the material [8,9]. Another strategy is incorporating secondary components such as rubbers [10,11], nanoparticles [12,13,14], the thermotropic liquid crystalline polymer [15,16,17], and thermoplastic resins [18,19,20,21] into epoxy resins. Unfortunately, adding rubber particles can significantly reduce the system’s modulus and glass transition temperature [22], and the uneven distribution of nanoparticles can lead to defects in the material [23]. In addition, the high cost and high processing temperature of thermotropic liquid crystals also limit their application in toughening modification.

In recent years, toughening and modification of epoxy resins using high-strength, high-toughness, high-heat-resistance thermoplastic resins have attracted the extensive attention of researchers [24,25]. Karthikeyan et al. [26] introduced hydroxyl-functionalized poly (ether ether ketone) (PEEK) into glycidyl ether of bisphenol A (DGEBA) epoxy resin, which improved its maximum lap shear strength and fracture toughness. Rehman et al. [27] investigated the effect of micro-PEEK particles on the damage response of carbon/epoxy composites under the low-velocity impact. They showed that introducing PEEK particles improved the toughness and damage tolerance of carbon/epoxy composites. Gresil et al. [4] used polyethersulfone (PESU) to modify epoxy anhydride thermosetting plastics and investigated its effect on the morphological, thermal, and mechanical properties of epoxy resins. Although the above thermoplastic resins enhanced the toughness of the epoxy resin, there was no significant improvement in its toughness due to their poor compatibility.

**Figure 1:**
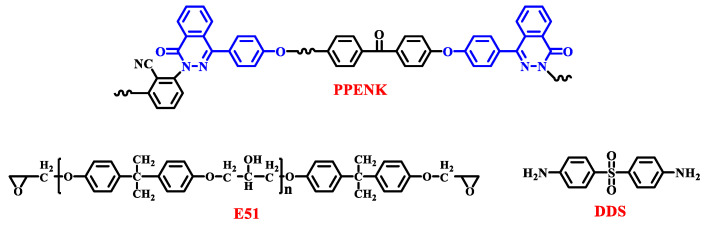
The molecular structure for PPENK, E51, and DDS.

Poly(phthalazinone ether nitrile ketone)s (PPENK, Figure 1), as a new type of high-performance engineering plastic, has excellent mechanical and thermal properties (glass transition temperature ≥ 240 °C) [28]. At the same time, it has a fully aromatic ring twisted non-coplanar molecular structure, giving it solubility. It can be hot-melt mixed with epoxy resin to improve its toughness and thermal properties.

Herein, we used the PPENK-containing phthalazinone structure to toughen epoxy resin. PPENK was hot-melt mixed with epoxy resin to prepare a series of epoxy blends with different contents of PPENK. The effects of PPENK content on the blends’ thermal properties, mechanical properties, and low-temperature properties were investigated. Therefore, this research is expected to open a new avenue for improving epoxy resin’s mechanical, thermal, and low-temperature properties using high-performance thermoplastic polymers.

## 2. Experiment

### 2.1. Materials and Methods

E51 epoxy (Figure 1) resin from Shenyang Haotian Resin Material Co., Ltd. (Shenyang, China), PPENK (Nitrile/Ketone, 1/1; Mn, 29,125 g/mol; density, 1.26 g/cm^3^; intrinsic viscosity, 0.46 dL/g) from Chengdu Tianshun Polymer New Material Co., Ltd., Liaoning, China, 4,4′-diaminodiphenyl sulfone (DDS, Figure 1) from Shanghai Maclean Biochemical Technology Co., Ltd. (Shanghai, China).

This study used the hot-melt mixed method to prepare the PPENK/E51 blends. The quality content of PPENK in the blend was 3, 5, 10, 15, and 20 phr (Parts per hundred parts of resin). The following is a brief description of the 10 phr sample.

A mass of 200 g of E51 and 10 g of PPENK were added to a three-necked flask, heated to 130 °C, and mixed for 1–2 h. A mass of 60 g of curing agent DDS was added, the solution was stirred well, and the hot mixture was poured into the mold (Figure S6) and placed in a vacuum oven to remove air bubbles. Then cured (procedure: 120 °C/2 h + 150 °C/1 h+ 190 °C/2 h + 220 °C/2 h).

### 2.2. Instrumentation and Methods

DSC 204 instrument tested the curing behavior of the uncured samples (5–10 mg) at different heating rates (β = 5, 10, 15, 20 °C/min) from 30 °C to 350 °C; and on blends (5–10 mg) to determine the glass transition temperature (*T_g_*) at a heating rate of 10 °C/min with a 3-stage ramp-up procedure: 30–250 °C, 250–30 °C, 30–250 °C. Additionally, the curing behavior of uncured samples (5–10 mg) was tested at a heating rate of 10 °C/min from 30 °C to 350 °C, and all tests were performed under an N_2_ atmosphere with a flow rate of 50 mL/min.

FT-IR spectra of the blends were obtained on a Nicolet-20DXB infrared spectrometer with the single reflection ATR method (range: 500 cm^−1^ to 4000 cm^−1^).

Mettler TGA/SDTA851 analyzer was used to test the thermal decomposition temperatures of the blends (5–10 mg) from 30 °C to 800 °C under N_2_ atmosphere (N_2_ flow rate of 50 mL/min) at a ramp rate of 20 °C/min.

The scanning electron microscope (SU8220 and FlexSEM 1000) was used to record the SEM images of the blend.

According to the ASTM D4812-99, the impact strength of non-notched samples with a dimension of 80 × 10 × 4 mm^3^ was measured from the XCJ-4 type Charpy machine, with at least 5 samples per group measured.

According to the ASTM D790-17, the flexural properties of samples with a dimension of 80 × 10 × 4 mm^3^ were tested by the Instron 5869 machine, with at least 5 samples per group measured.

According to the ASTM D638-22, the tensile properties of the samples with a dimension of 115 × 10 × 4 mm^3^ (dumbbell shape) were tested by the Instron 5869 machine, with at least 5 samples per group measured.

According to the ASTM D790-17, the samples’ low temperatures (−183 °C) flexural properties with a dimension of 80 × 10 × 4 mm^3^ were tested by the SDS-100 machine fitted with a thermodynamic environmental chamber, with no less than 5 samples per group.

According to the ASTM D638-22, the low temperatures (−183 °C) tensile properties of the samples with a dimension of 115 × 10 × 4 mm^3^ (dumbbell shape) were tested by the SDS-100 machine fitted with a thermodynamic environmental chamber, with at least 5 samples per group measured.

TA Q800 dynamic mechanical analyzer (single cantilever mode, the frequency was 1 HZ) was used for testing the dynamic thermomechanical properties of 40 × 6 × 3 mm^3^ rectangular samples at 5 °C/min from 35 °C to 250 °C.

## 3. Results and Discussion

### 3.1. Curing Behavior

**Figure 2:**
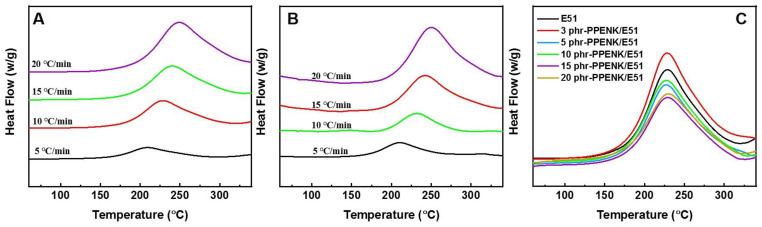
DSC curves: (**A**) E51 and (**B**) 10 phr-PPENK/ E51 (different heating rates), (**C**) PPENK/E51 blends with different content.

The curing behavior of neat E51 and 10 phr-PPENK/E51 blend was investigated to demonstrate the effect of PPENK on E51 resin’s curing kinetics. Figure 2A,B are the DSC curves of neat E51 and 10 phr-PPENK/E51 blends with different ramp rates, respectively. The heating curve of all systems exhibited a single and symmetrical feature. In addition, as the heating rate increased, the exothermic peak became higher and steeper. It shifted towards high temperatures direction, mainly due to the significant temperature difference in the system. The apparent activation energy (Ea) is one of the most important parameters for measuring the curing reaction activity of the systems [29]. It can usually be calculated by the Kissinger and the Flynn–Wall–Ozawa equations (Supplementary Material) [30], and the values of Ea are shown in Table 1. Ea of 10 phr-PPENK/E51 blends were slightly different from the pure E51, suggesting that PPENK had a smaller influence on the curing reaction of E51. In addition, Figure 2C shows the DSC curves (at a heating rate of 10 °C/min) for the different contents of PPENK blends. All curves showed only one exothermic peak, and PPENK did not increase the exothermic peak, indicating that temperature was decisive in the curing process and that the influence of PPENK content was small.

### 3.2. FT-IR Analysis

**Figure 3:**
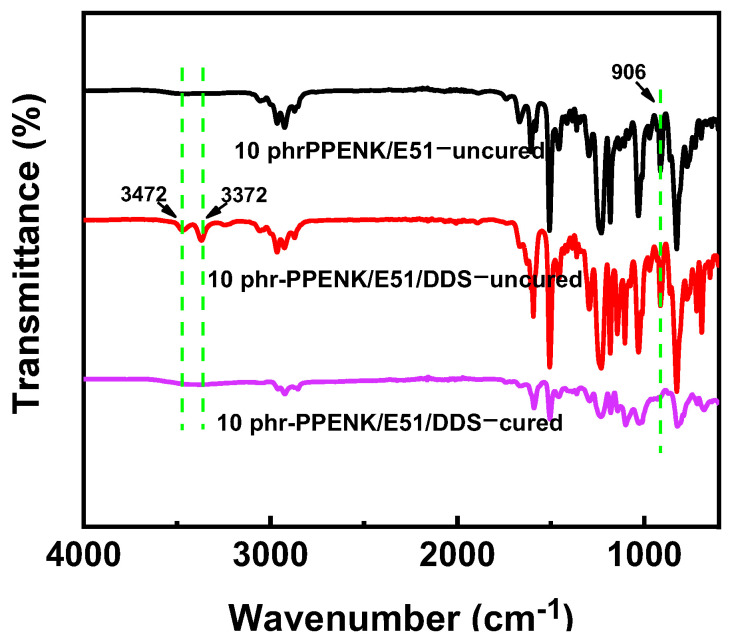
FTIR of 10 phr−PPENK/E51 blend with or without curing.

To clarify the influence of PPENK on the chemical structure of cured E51 resin. FT-IR of 10 phr-PPENK/E51 was demonstrated in Figure 3. As seen in Figure 2, 906 cm^−1^ was the characteristic absorption peak of the epoxy group [31], and 3372 cm^−1^ and 3472 cm^−1^ were assigned to the characteristic peaks of N-H in the curing agent DDS. The above characteristic peaks disappeared completely after curing, indicating that the blends could be cured adequately under the curing procedure (120 °C/2 h + 150 °C/1 h+ 190 °C/2 h + 220 °C/2 h).

### 3.3. Glass Transition Temperatures (T_g_)

**Figure 4:**
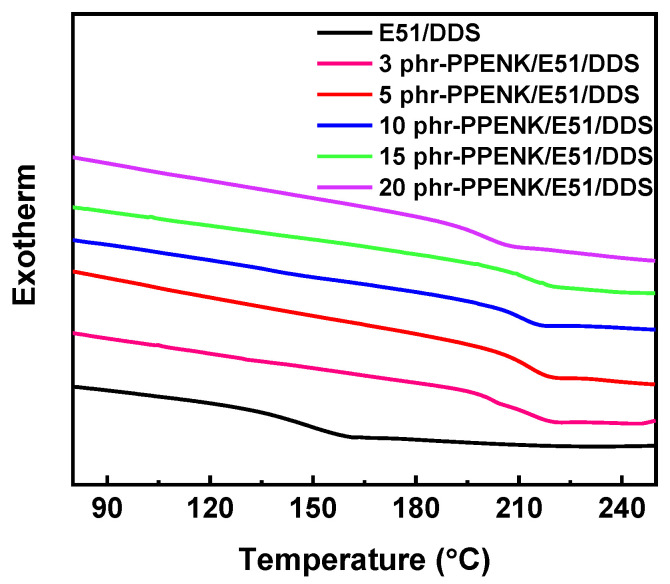
DSC curves of the blends with or without PPENK.

DSC tested the *T_g_* of blends with or without PPENK to determine the impact of PPENK on the thermal characteristics of E51. As shown in Figure 4, the *T_g_* of the blends was significantly improved after the introduction of PPENK. The *T_g_* of the PPENK/E51 blends were summarised in Table 2. On the one hand, PPENK had a higher *T_g_* than E51 (see Table S4). Thus it would increase the *T_g_* of the blend system. On the other hand, the introduction of PPENK caused a gradual decrease in the blend system’s crosslink density (Table S3) [32], leading to the blend’s *T_g_* decrease. The combination of these two effects made the *T_g_* of the blend system increase first and then decrease.

### 3.4. Thermomechanical Properties

**Figure 5:**
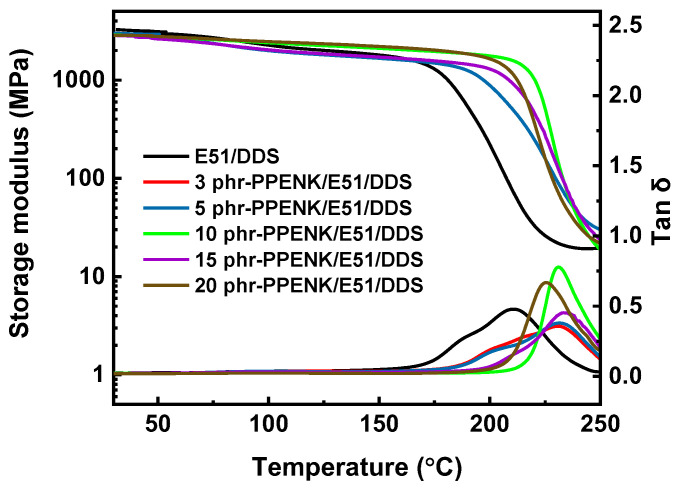
Thermomechanical properties of the blends.

The storage modulus (Er) and loss factor (Tanδ) of the cured E51 systems are depicted in Figure 5. From Figure 5, the storage modulus of the blends decreased slowly with increasing temperature, and the storage modulus decreased sharply when the temperature continued to increase, mainly due to the transition from the glass state of the system to the high elastic state. Interestingly, the initial-Er of the blends containing PPENK decreased compared with E51 due to a lower crosslink density of E51 after the addition of PPENK. On the one hand, the Er of the blend system mainly depended on the flexibility of the molecular segments [33]. The greater the flexibility, the lower the storage modulus. In the glass state, pure E51 had a large crosslink density, the molecular chain formed a network, and the flexibility of the molecular chain segment was poor, so the storage modulus was high [34]. After the introduction of PPENK, the PPENK/E51 blend’s crosslink density decreased, and the flexibility of the molecular segments increased, resulting in a decrease in the initial Er. The effect of PPENK on the blend’s crosslink density was explored using the theoretical equations of rubber elasticity (Supplementary material). The values of crosslink density are listed in Table S3. From Table S3, as PPENK content increased, the crosslink density of the blends gradually reduced; this was attributable to the dilution effect caused by adding PPENK, which decreased the density of reactive groups per unit volume, thereby reducing the crosslinking density [35,36].

The inflection point temperature of the polymer storage modulus curve was usually considered to be the *T_g_* of the polymer. The *T_g_* of the blended system with PPENK was significantly increased, similar to the DSC test results. In addition, there was only one loss peak in all blends, which indicated that the blend was a homogeneous system without interfacial separation.

### 3.5. Mechanical Properties

**Figure 6:**
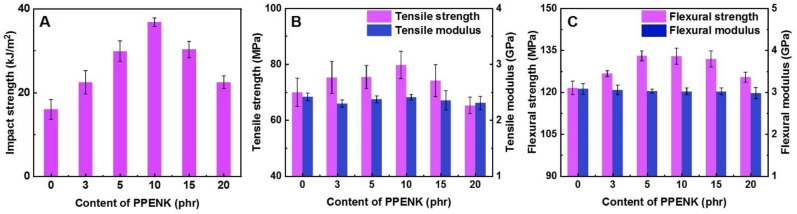
Mechanical properties of the blends: (**A**) Impact strength, (**B**) Tensile properties, (**C**) Flexural properties.

The mechanical properties of different proportions of PPENK/E51 blends are shown in Figure 6. As shown in Figure 6A, the impact strength of the blend system gradually increased as PPENK content was raised. When the content was 10 phr, the impact strength of the system reached a maximum of 37.0 kJ/m^2^, which was about 131.0% higher than that of pure E51. When PPENK increased to 15 and 20 phr, the impact strength of the system decreased. In addition, as shown in Figure 5B,C, the changing trend of tensile and flexural strength was comparable to that of impact strength. When the content was 10 and 15 phr, the tensile and flexural strength reached the maximum value of 80 MPa and 133 MPa, respectively; this presented that PPENK could effectively increase the toughness and strength of E51 resin. For comparison, Table 3 shows some of the previous reports.

**Figure 7:**
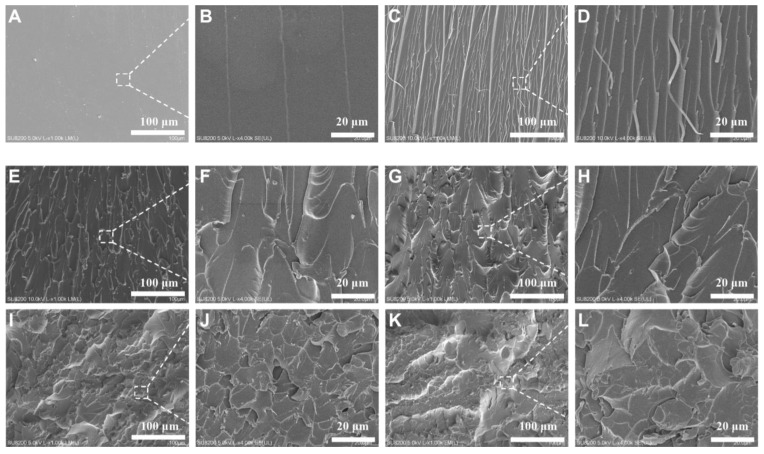
SEM micrographs of the blend for impact fractures ((**A**,**B**) E51, (**C**,**D**) 3 phr, (**E**,**F**) 5 phr, (**G**,**H**) 10 phr, (**I**,**J**) 15 phr, (**K**,**L**) 20 phr).

SEM observed the fracture surface after impact fracture to understand the toughening mechanism of PPENK−modified E51. From Figure 7A,B, pure E51 had a smooth fracture surface, the crack direction was single, and there was no apparent stress dispersion. On the contrary, after the introduction of PPENK in E51, the fracture morphology of the blend changed from smooth to relatively rough, and the cracks showed noticeable deflection and disproportionation. In addition, as shown in Figure 7H–L, there were many micro-cracks on the fracture surface, which increased the area of the fracture surface, and an apparent stress dispersion phenomenon occurred, which increased the fracture energy [37]. In addition, there were many polar groups (-CN) in the molecular chain of PPENK. These polar groups made a large number of interactions between PPENK and E51 [32]. When an external force damages the blend system, these weak interactions can withstand stress, consuming more crack propagation energy [38]. In addition, it has been reported in the literature that the blend system could exhibit a large amount of plastic deformation during the fracture process, thereby improving the toughness of the blend system [39].

**Figure 8:**
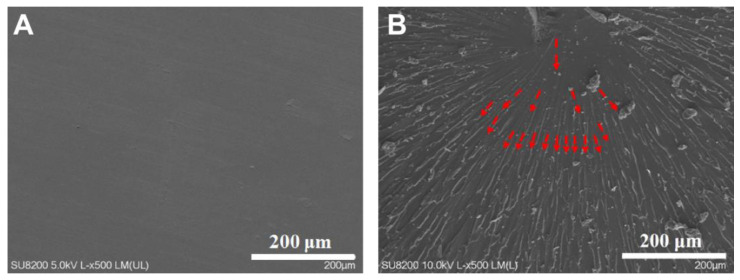
SEM micrographs and crack propagation path of 10 phr-PPENK/E51 blend ((**A**) Before fracture, (**B**) After fracture).

Figure 8 shows the SEM of the 10 phr-PPENK/E51 blend before and after fracture, as well as a schematic diagram of crack extension. The pure E51 system had a high cross-linking density, while the cross-linked network had poor resistance to crack propagation, which was prone to crack and brittle fracture. The introduction of PPENK increased the resistance of PPENK/E51 blends to crack propagation. In Figure 8B, the crack gradually spreads from the impact point to the surroundings. It was continuously deflected, increasing the energy consumption of crack propagation [39,40]. Therefore, it could be speculated that PPENK toughens E51 mainly through crack deflection, which was consistent with the previous conclusion.

### 3.6. Low−Temperature Properties

**Figure 9:**
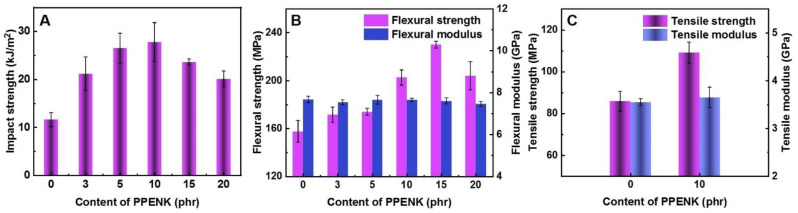
Mechanical properties of cured PPENK/E51 blends at low-temperature (−183 °C): (**A**) Impact strength, (**B**) flexural properties, (**C**) tensile properties.

Low−temperature performance was one of the important physical properties of epoxy resin. In this section, the evolution law of mechanical properties of epoxy resins in the liquid-oxygen temperature range was studied, and the corresponding results are shown in Figure 9. In the low-temperature environment (−183 °C), the epoxy resin also bears the internal shrinkage force while the external force is applied. In addition, the degree of molecular orientation of E51 resin increased results in smaller free volumes and stress concentration aggravated, making it easier to form cracks and brittle fractures when impacted [41]. Therefore, its impact strength at low-temperature values was generally low (Figure 9A). When PPENK was introduced, the polar molecules of PPENK made it compatible with E51, forming a uniform interpenetrating structure, which expanded the distance between E51 molecules and increased the activity space of E51 molecules, causing the PPENK/E51 blends to have some flexibility at low temperature (−183 °C). In addition, the PPENK molecule contained a twisted, non-coplanar bis-diazapine structure [42,43]. This unique structure enabled the molecular chain to maintain a sizeable free volume even at low temperatures, and its toughness can be well genetically preserved. The above two worked together to make the blend keep good impact toughness even at low-temperature (−183 °C).

The flexural properties of PPENK/E51 blends were also significantly affected in low-temperature environments. Based on Figure 9B, the flexural strength and modulus of pure E51 and PPENK/E51 blends had significantly improved. When the content of PPENK was 15 phr, the maximum bending strength was 230 MPa, which was 45.6% and 88.5% higher than pure E51 at low-temperature (−183 °C) and room temperature, respectively. In low-temperature environments, the molecular chains of E51 and PPENK shrink and become rigid, and the overall stiffness of the system increases [44,45]. In addition, reducing the intermolecular distance increased the intermolecular force, increasing the frictional resistance between the molecules, so the load at the time of failure was larger. Therefore, the PPENK/E51 blend’s flexural strength and modulus were increased.

Similar to the flexural properties at low-temperature (−183 °C), the PPENK/E51 blend’s tensile strength and modulus were improved compared to those at room temperature. Figure 9C shows the low-temperature tensile strength of PPENK/E51 blends. The 10 phr-PPENK/E51 blend’s tensile strength was 109 MPa, 33.8% higher than at room temperature (80 MPa, Figure 6B). At the low temperature (−183 °C), the molecular chains of the blends were frozen, and the resin fluidity was reduced, resulting in tensile strength and modulus increase [46].

**Figure 10:**
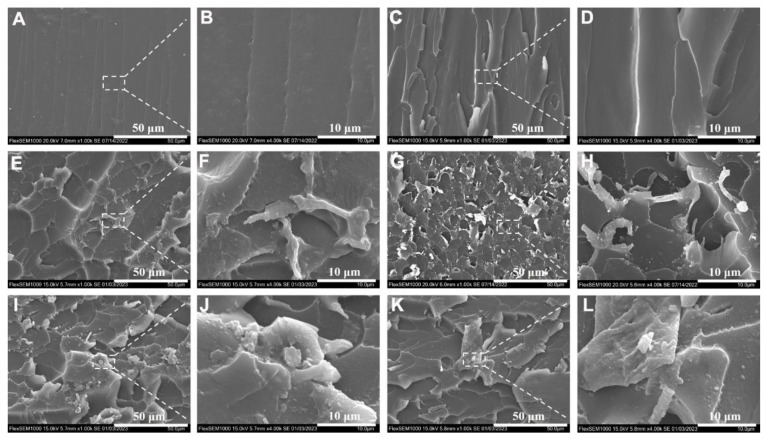
SEM micrographs of the low-temperature impact section of PPENK/E51 ((**A**,**B**) E51, (**C**,**D**) 3 phr, (**E**,**F**) 5 phr, (**G**,**H**) 10 phr, (**I**,**J**) 15 phr, (**K**,**L**) 20 phr).

Figure 10 shows the blend’s SEM image after the low-temperature (−183 °C) impact fracture. From Figure 10, the 10 phr−PPENK/E51 blend’s fracture surface was rougher and had more microcracks than the pure E51 resin. On the one hand, PPENK and E51 blended uniformly during molding. As a stress concentration point, PPENK could induce more microcracks to absorb energy and delay the propagation of cracks when stressed. On the other hand, PPENK riveted the damage by bridging it in the E51 matrix to restrain crack propagation. Combining these two effects increased the impact toughness of the 10 phr-PPENK/E51 blend.

### 3.7. Thermal Stability

**Figure 11:**
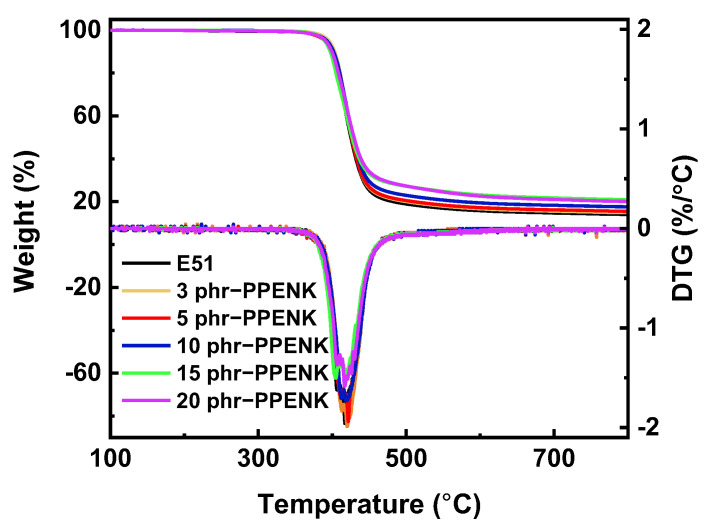
TGA curves of PPENK/E51 blends.

Thermogravimetric analysis (TGA) was used to test the thermal stability of the cured blend systems [47,48], and the thermal weight loss curves were displayed in Figure 11. In addition, the blend’s 5% thermal weight loss temperature (*T_d5%_*) and maximum thermal weight loss temperature (*T_dmax_*) are shown in Table 4. From Table 4, the PPENK/E51 blends all had a *T_d5%_* above 390 °C and a *T_dmax_* of around 420 °C. Compared with pure E51, these values did not change much, and some increased. Interestingly, the residual mass at 800 °C increased with increasing PPENK content, which was attributed to the excellent thermal properties of PPENK (Table S3). Therefore, although the addition of PPENK led to a decrease in the crosslink density of E51, the good thermal properties of PPENK made up for this shortcoming, so PPENK/E51 still had good thermal stability.

## 4. Conclusions

High-performance thermoplastic poly aryl ether PPENK was applied to modify the epoxy resin E51. With the addition of PPENK, the crosslink density of E51 was reduced. At the same time, the *T_g_* of the blends was significantly increased (~50 °C). The blends had excellent mechanical and low−temperature properties (−183 °C). When the PPENK content was 10 phr, the impact strength of the blend was 131.0% higher than pure E51. When the PPENK content was 15 phr, the low−temperature flexural strength was 46.5% higher than pure E51. All in all, this research will provide a fresh perspective and theoretical support to improve epoxy resins’ mechanical, thermal, and low-temperature properties.

## Figures and Tables

**Table 1:** The DSC dates of the different blends.

Blend Systems	Ea by Kissinger Equation (kJ/mol)	Ea by Ozawa Equation (kJ/mol)	Ea Average (kJ/mol)	*T_i,β_* = 0 (°C)	*T_top,β_* = 0 (°C)	*T_f,β_* = 0 (°C)
E51/DDS	52.6	67.4	60.0	134	201	248
10 phr-PPENK/E51	50.4	65.4	57.9	129	200	241

**Table 2:** The *T_g_* and thermomechanical properties of blends.

Blend Systems	*T_g_* (°C)		Initial-Er
	DSC	DMA	(GPa)
E51	154	166	3.27
3 phr-PPENK/E51	208	186	3.15
5 phr-PPENK/E51	214	188	3.00
10 phr-PPENK/E51	211	206	2.90
15 phr-PPENK/E51	213	201	2.88
20 phr-PPENK/E51	200	198	2.87

**Table 3:** Comparison of the toughening effect of PPENK/EP blend composites with the literature.

Blends	Content	Improvement Rate	Ref.
GO/PSF/EP	5.2 phr	89.9%	[14]
LCP/EP	10 phr	30%	[16]
DGE-DHBP/EP	10%	42.2%	[17]
PKHH/EP	10%	81.5%	[18]
PES/PEK−C/EP	15%	99.8%	[19]
PET/EP	4.8%	30.0%	[21]
PPENK/EP	10 phr	131.0%	This work

**Table 4:** Thermal stability date of PPENK/E51 blends.

Blend Systems	*T_d5%_* (°C)	*T_dmax_* (°C)	Char Yield (%)
E51	392	417	8.35
3 phr−PPENK/E51	395	420	12.99
5 phr−PPENK/E51	391	421	14.95
10 phr−PPENK/E51	393	418	17.97
15 phr−PPENK/E51	390	417	17.19
20 phr−PPENK/E51	391	417	20.54

## Data Availability

Not applicable.

## Supplementary Materials

The following supporting information can be downloaded at: https://www.mdpi.com/article/10.3390/ma16072878/s1, Figure S1: Curves of PPENK/E51 blends curing exothermic peak temperature and heating rate: A E51, B 10 phr-PPENK/E51. Figure S2: Fitting line of E51/DDS: A Kissinger method, B Ozawa method; Figure S3: Fitting line of 10%-PPENK/ E51/DDS: A Kissinger method, B Ozawa method; Figure S4: A DSC curves and B TGA curves of PPENK. Figure S5: A Typical tensile load–displacement curves, B Typical flexural load–displacement curves; Figure S6: Curing molds for PPENK/E51 blends; Table S1: Curing exothermic peak temperature of E51 at different heating rates. Table S2: Curing exothermic peak temperature of PPENK/E51 at different heating rates. Table S3: The crosslinking density of different blend systems. Table S4 DSC data and TGA data of PPENK; Table S5: The mechanical properties of blends at room temperature and ultra-low temperature.
