# Supplementary material for: A New Strategy to Improve the Toughness of Epoxy Thermosets—By Introducing Poly(ether nitrile ketone)s Containing Phthalazinone Structures
Source: Materials (Basel). 2023 Apr 4;16(7):2878. doi: 10.3390/ma16072878 (PMC10096459; doi:10.3390/ma16072878)
Supplement: Supplementary file 1 [file materials-16-02878-s001.zip › materials-2286170-supplementary.pdf]

Supplementary material

A new strategy to improve the toughness of epoxy thermosets—by introducing poly(phthalazinone ether nitrile ketone)s containing phthalazinone structure

Hongjun Guo [a,b,d], Bing Wang [a,b,c], Xin Fu [e], Nan Li [a,b]*, Guiyang Li [d], Guodong Zheng [d], Zaiyu Wang [f], Cheng Liu [a,b], Yousi Chen [a,b], Zhihuan Weng [a,b], Shouhai Zhang [a,b], Xigao Jian [a,b,c]*

[a] *State Key Laboratory of Fine Chemicals, Frontier Science Center for Smart Materials, Dalian University of Technology, Dalian 116024, China;*

[b] *Technology Innovation Center of High Performance Resin Materials, Liaoning Province, 116024, People's Republic of China;*

[c] *School of Materials Science and Engineering, Dalian University of Technology, Dalian 116024, China;*

[d] *Aerospace Research Institute of Materials & Processing Technology, Beijing, 100076, China;*

[e] *Wuhan Second Ship Design and Research Institute, Wuhan, 430064, China;*

[f] *AVIC Jiangxi Hongdu Aviation Industry Group Company Ltd, Nanchang, China.*

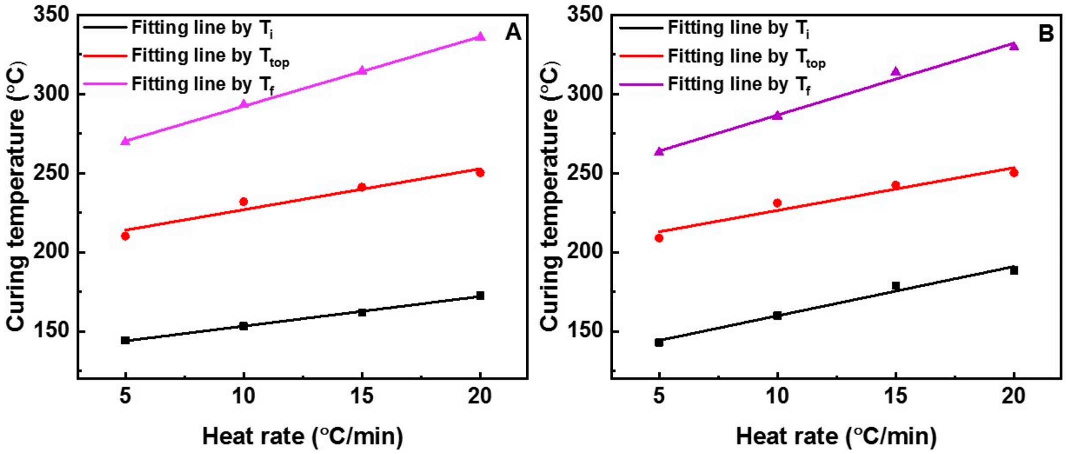

Figure S1 Curves of PPENK/E51 blends curing exothermic peak temperature and heating rate: A E51, B 10 phr-PPENK/E51.

Table S1 Curing exothermic peak temperature of E51 at different heating rates.

Heat rate (°C/min)	T_i (°C)	T_{top} (°C)	T_f (°C)
5	144	210	270
10	153	232	293
15	162	241	315
20	173	250	336

Table S2 Curing exothermic peak temperature of PPENK/E51 at different heating rates.

Heat rate (°C/min)	T_i (°C)	T_{top} (°C)	T_f (°C)
5	143	209	263
10	160	231	286
15	179	242	314
20	189	250	330

Table S3 The crosslinking density of different blend systems.

Blend systems	T_g (°C)	Er (MPa)	crosslinking density(mol/m^3)
E51/DDS	166	32.7	2.80×10^3
3%-PPENK/E51/DDS	186	30.6	2.51×10^3
5%-PPENK/E51/DDS	188	27.6	2.21×10^3
10%-PPENK/E51/DDS	206	23.61	1.86×10^3
15%-PPENK/E51/DDS	201	11.8	0.94×10^3
20%-PPENK/E51/DDS	198	8.8	0.89×10^3

Calculation of crosslinking density of blends

The crosslinking density of E51 and 10%-PPENK/E51 were calculated jointly by theoretical formula of rubber elasticity:

$$E_r = 3\Phi RT$$

where E_r is the storage modulus (MPa), Φ is the crosslinking density(mol/m^3), R is the gas constant (8.314 J/mol·K), T= T_g+30°C.

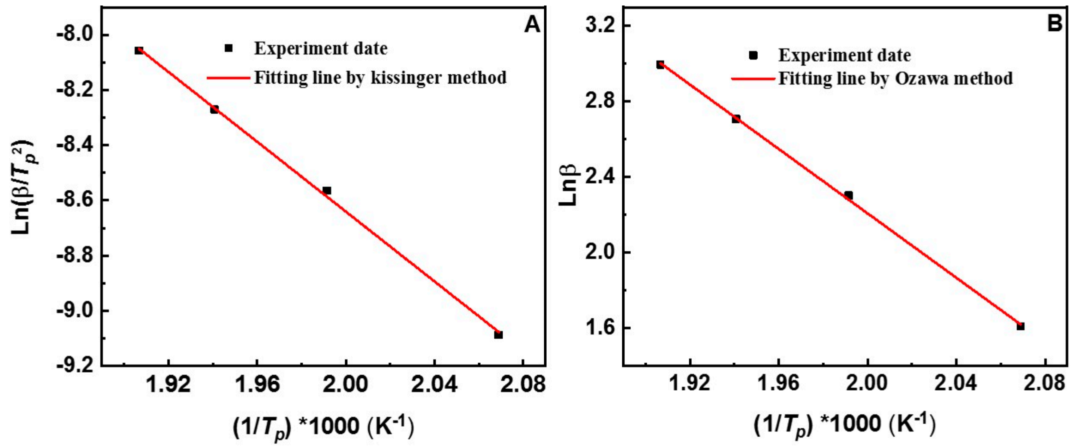

Figure S2 Fitting line of E51/DDS: A Kissinger method, B Ozawa method

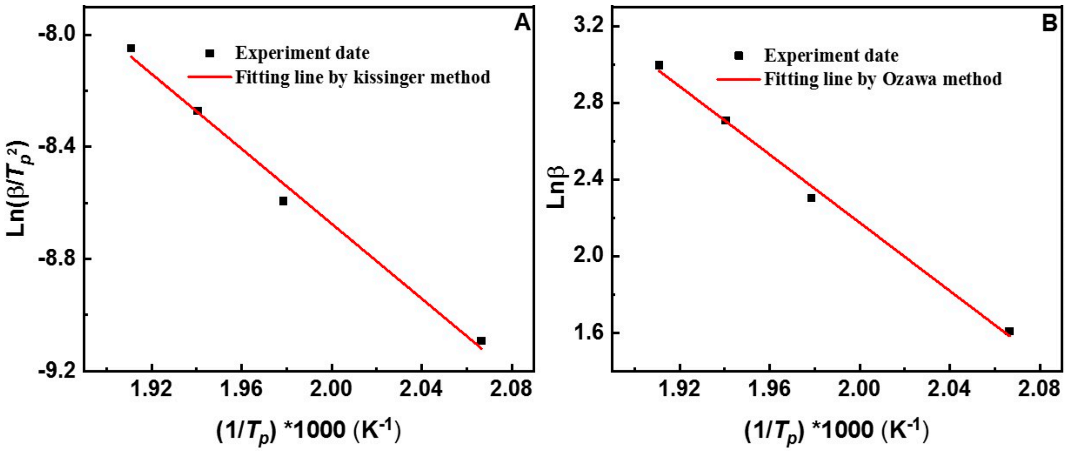

Figure S3 Fitting line of 10%-PPENK/ E51/DDS: A Kissinger method, B Ozawa method

Calculation of activation energy of blends

The apparent reaction activation energy of E51 and 10%-PPENK/E51 were calculated jointly by Kissinger method and Ozawa method, Kissinger equation is as follows:

$$\frac{d\ln\left(\beta/T_p^2\right)}{d\left(1/T_p\right)} = -\frac{\Delta E_a}{R} + \ln\left(\frac{AR}{E_a}\right) \tag{S1}$$

where β is the heating rate (K/min), T_P is the peak top temperature on the DSC curves (K), R is the gas constant (8.314 J/mol·K), and E_a is the reaction activation energy (KJ/mol).

Ozawa equation is as follows:

$$\frac{d\ln\beta}{d\left(1/T_P\right)} = -1.052\frac{\Delta E_a}{R} \tag{S2}$$

where β is the heating rate (K/min), T_P is the peak top temperature on the DSC curves (K), R is the gas constant (8.314 J/mol·K), and E_a is the reaction activation energy (KJ/mol).

As shown in Figure S2-S3, the linear fitting is carried out by $\ln\left(\beta/T_p^2\right)-\left(1/T_p\right)\times10^3$ and $\ln\left(\beta\right)-\left(1/T_p\right)\times10^3$ data, respectively. The average activation energy was calculated based on slopes of the straight lines, as shown in table 1.

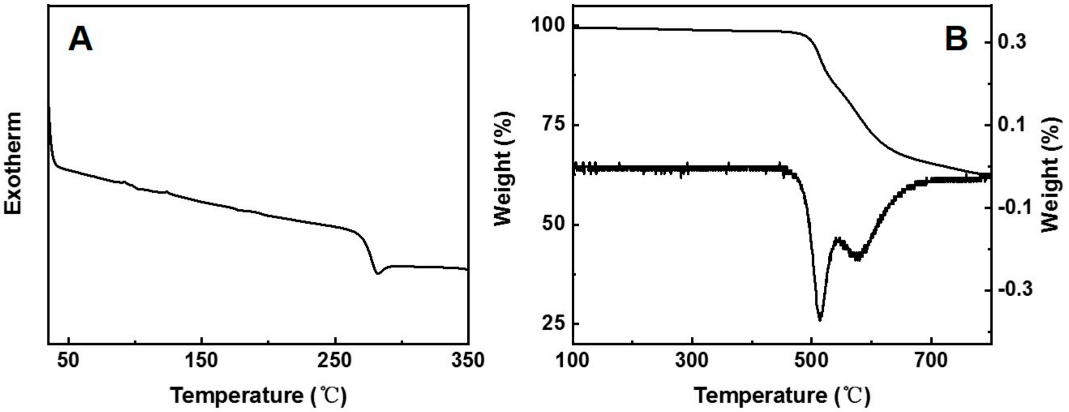

Figure S4 A DSC curves and B TGA curves of PPENK.

Table S4 DSC data and TGA data of PPENK

Polymer	T_g (°C)	$T_{d5\%}$(°C)	T_{dmax}(°C)	Char yield (%)
PPENK	276	505	515	62

Table S5 The mechanical properties of blends at room temperature and ultra-low temperature

Blend systems	Impact strength (kJ/m^2)		Flexural strength (MPa)		Tensile strength (MPa)	
	RT	ULT	RT	ULT	RT	ULT
0%	16.0 ± 2.3	11.6 ± 1.4	121 ± 2	157 ± 9	70 ± 5	86 ± 5
3%	22.4 ± 2.8	21.2 ± 3.5	127 ± 1	171 ± 6	75 ± 6	---
5%	29.9 ± 2.4	26.5 ± 3.1	133 ± 2	174 ± 3	75 ± 4	---
10%	37.0± 1.0	27.8 ± 4.1	133 ± 3	202 ± 6	80 ± 5	110 ± 5
15%	30.3 ± 2.0	23.6 ± 0.6	132 ± 3	230 ± 3	74 ± 6	---
20%	22.5 ± 1.5	20.1 ± 1.7	125 ± 2	204 ± 11	65 ± 3	---

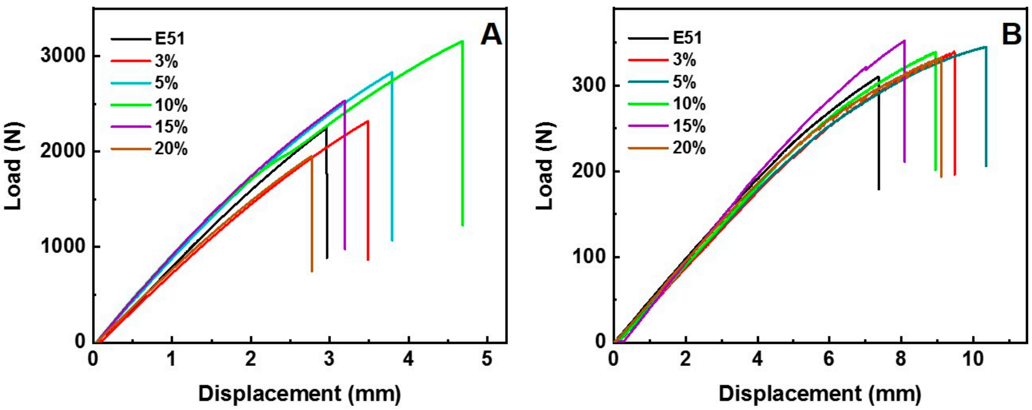

Figure S5 **A** Typical tensile load–displacement curves, **B** Typical flexural load–displacement curves.

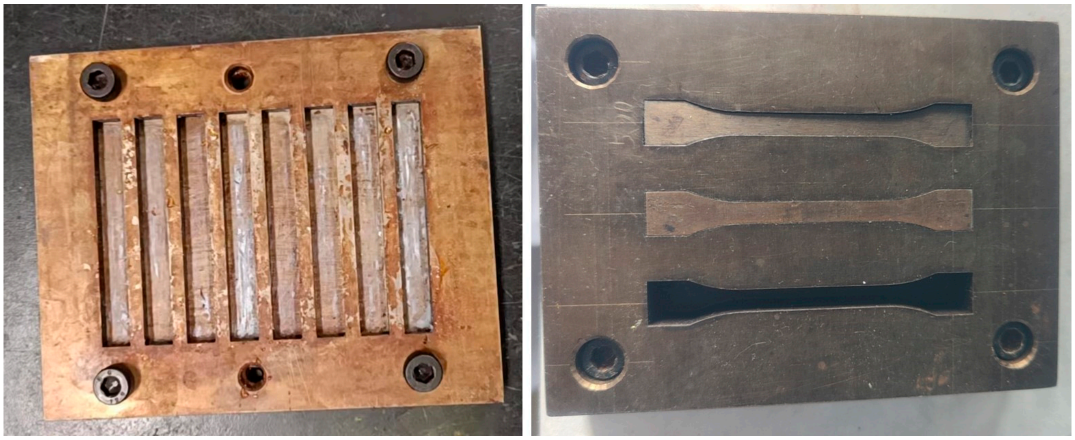

Figure S6 Curing molds for PPENK/E51 blends
